# *Lacticaseibacillus rhamnosus* GG Counteracts Rotavirus-Induced Ion Secretion and Enterocyte Damage by Inhibiting Oxidative Stress and Apoptosis Through Specific Effects of Living and Postbiotic Preparations

**DOI:** 10.3389/fcimb.2022.854989

**Published:** 2022-03-29

**Authors:** Vittoria Buccigrossi, Marco Poeta, Valentina Cioffi, Sara Terranova, Francesco Nunziata, Andrea Lo Vecchio, Alfredo Guarino

**Affiliations:** Department of Translational Medical Science, Section of Pediatrics, University of Naples Federico II, Naples, Italy

**Keywords:** rotavirus, *Lacticaseibacillus rhamnosus* GG, gastroenteritis, diarrhea, enterocyte damage, oxidative stress, probiotics, postbiotics

## Abstract

**Background:**

Administration of *Lacticaseibacillus rhamnosus* GG (LGG) to children with gastroenteritis is recommended by universal guidelines. Rotavirus (RV) causes diarrhea through combined cytotoxic and enterotoxic effects. Aim of this study was to evaluate the mechanisms of efficacy of LGG in an *in-vitro* model of RV diarrhea in its viable form (LGG) and conditioned medium (mLGG).

**Methods:**

Ion secretion corresponding to the NSP4 enterotoxic effect, was evaluated by short circuit current (*Isc*) and the cytotoxic effect by transepithelial electrical resistance (TEER) in Ussing chambers, upon exposure to RV in Caco-2 enterocyte monolayers treated or not with living probiotic or its culture supernatant. Mechanisms of enterotoxic and cytotoxic damage were evaluated including oxidative stress measured by reactive oxygen species, apoptosis evaluated by DAPI and nuclear staining, NFkβ immunofluorescence.

**Results:**

RV induced *Isc* increase and TEER decrease, respectively indicating ion secretion and epithelial damage, the two established pathways of diarrhea. Both probiotic preparations reduced both diarrheal effects, but their potency was different. Live LGG was equally effective on both enterotoxic and cytotoxic effect whereas mLGG was highly effective on ion secretion and showed minimal protective effects on cytoskeleton, apoptosis and NFkβ.

**Conclusions:**

LGG counteracts RV-induced diarrhea by inhibiting both cytotoxic and enterotoxic pathogenic mechanisms. Namely, LGG inhibits chloride secretion by specific moieties secreted in the medium with a direct pharmacologic-like action. This is considered a postbiotic effect. Subsequently, live bacteria exert a probiotic effect protecting the enterocyte structure.

## Introduction

Specific probiotics have been recommended as adjunctive treatment of gastroenteritis by several guidelines all over the world (Guarino et al., 2018). Many clinical trials showed that diarrhea is rapidly reduced upon administration of *Lacticaseibacillus rhamnosus* GG (LGG), and the effects are already observed within hours after the onset of therapy (Guarino et al., 2015). However, the mechanisms of action of LGG are not entirely clear. An established long-term effect by LGG is the restoration of microbiota in children. This was supported by several papers in healthy subjects (Cox et al., 2010; Rauch and Lynch, 2010) and in cystic fibrosis children (Bruzzese et al., 2014). However, this effect does not explain the rapid efficacy of LGG on diarrhea, which is already observed within hours after administration.

Rotavirus (RV) infection is the most frequent and severe form of acute gastroenteritis in infants and children worldwide (Lo Vecchio et al., 2017). RV severity is related to a combination of time-related mechanisms leading to secretory and osmotic diarrhea through a sequence of molecular events (De Marco et al., 2009). In the early phase of infection, RV directly induces active chloride and water secretion from the enterocyte into the intestinal lumen through the enterotoxic effects of the non-structural viral protein NSP4. This increases intracellular Ca^2+^ concentration which in turn triggers electrogenic chloride secretion (De Marco et al., 2009; Ousingsawat et al., 2011; Buccigrossi et al., 2014). Oxidative stress is a key mechanism involved in the enterotoxic effect induced by RV (Buccigrossi et al., 2014).

Following early ion secretion, RV infection results in severe damage to the structure of intestinal villi with cell death and subsequent disruption of epithelial integrity (Medici et al., 2011) whose clinical expression is osmotic diarrhea consequent to malabsorption of nutrients which triggers an increased passive flux of water into the intestinal lumen.

The key treatment of acute gastroenteritis in children is the administration of oral rehydration solution (ORS) (Guarino et al., 2014) but this neither shortens the duration of diarrhea nor reduces the frequency of stool output. Therefore, additional therapies are recommended in adjunct to ORS in order to reduce intensity and duration of the disease. Administration of selected probiotics, including LGG and *Saccharomyces boulardii*, is the main approach to achieve such effect (Guarino et al., 2014).

*In-vitro* and *in-vivo* studies indicate that *S. boulardii* exerts its antidiarrheal effect acting on the resident microflora and inducing an anti-inflammatory effect (Pothoulakis, 2009). However its rapid antidiarrheal effect might be due to secreted molecules directly acting on intestinal epithelial cells inhibiting the secretive diarrhea through an anti-oxidant mechanism (Buccigrossi et al., 2014). This is defined as “postbiotic effect” (Tsilingiri and Rescigno, 2013) and was proposed for several bacteria (Levy et al., 2015), including LGG (Cicenia et al., 2016; Gao et al., 2019).

In clinical trials LGG reduces secretory diarrhea in very short time, measurable in hours. This suggests that neither changes in microflora (Cox et al., 2010) nor anti-inflammatory effect (Pagnini et al., 2018) are implicated with such the rapid efficacy. LGG directly interacts with intestinal epithelial cells but the exact mechanisms of diarrhea reduction it is not clear. In the present study, we investigated the effects of LGG in either form of living bacteria and LGG-conditioned medium on specific RV-induced enterotoxic and cytotoxic effect in our experimental model of human derived intestinal epithelium.

## Materials and Methods

### Cell Line

Caco-2 cells (American Type Culture Collection, Middlesex, UK) were used as a model of mature and differentiated enterocytes. Cells were grown in high glucose DMEM with 10% fetal calf serum (FBS), 1% non-essential amino acids, 50 mg/ml streptomycin, 50mU/ml penicillin. The cells were grown for 15-18 days after confluence on polycarbonate Snapwell filters (pore size 0,4 micron) (Costar Italia, Milan, Italy).

### Virus Strain and Infection Protocol

The infection of Caco-2 cell monolayers was performed with the simian rotavirus strain SA11 (RV) at a multiplicity of infection (MOI) of 25. RV activation was performed with 20 µg/mL trypsin for 1 hour at 37°C. Then, viral sample was added to the apical side of the Caco-2 cell monolayers for 1 hour at 37°C, then the cells were rinsed 3 times and incubated in serum–free medium for 1 hour. Then, Caco-2 cells were mounted in Ussing chamber system as described below. This model has been currently used to test the effects of antimicrobial molecules (Buccigrossi et al., 2014) and has become a standard tool for studying the direct effects of drugs and toxins in human intestinal epithelial cells. Cells were incubated with LGG in different conditions as described below before and after RV infection.

### Preparation of *Lacticaseibacillus* Culture Supernatant

A preparation of LGG supernatant (6x10^9^ u.f.c.). LGG were grown in Dulbecco**’**s modified Eagle essential medium (DMEM; Life Technologies Italia, Monza, Italy) with a high glucose concentration (4.5 g/L); 10% fetal bovine serum (FBS, Life Technologies Italia, Monza, Italy) and 1% non-essential amino acids were added. LGG were cultured for 72 h at 42°C. The cells-free culture supernatant (mLGG) was obtained by centrifugation and the subsequent passage through a 0.22 mm filter. The stock used in this study has a protein content of 1.5 mg/mL

### Ion Transport Studies

The Ussing chamber system is used to evaluate ion transport in polarized cell monolayers, grown on permeable supports. Caco-2 cells were short-circuited by a voltage clamp in Ussing chambers (Physiological Instruments, San Diego, CA). Electrical parameters analyzed are: short circuit current [*Isc* represents the short circuit required to bring Vt to 0 mV expressed in µA/cm^2^, epithelial membrane resistance expressed as tissue ionic conductance (G measured in mS/cm^2^) and, finally, transmembrane voltage (Vt, expressed in mV)]. Every 20 sec current pulses were passed across the epithelium and the current was measured and transepithelial resistance (R) was calculated.

*Isc* is the parameter that is a measure of the secretory/absorptive result. The integrated response was calculated between 10 and 50 mins. In addition, the peak of *Isc* response obtained in the time period considered (after subtraction of baseline *Isc*; named Δ *Isc*) was used as a measure of the secretory/absorptive response. The proabsorptive effect is indicated as negative value of Δ *Isc* whereas the positive values indicate an active ion secretion. The maximal increase of *Isc* (Δ *Isc*) is considered the peak effect (Buccigrossi et al., 2020). Caco-2 cell monolayers were stimulated with LGG or mLGG 1 hour before RV infection and for the duration of the experiment.

### Reactive Oxygen Species (ROS) Production Assay

DCFH-DA spectrofluorometry was used to measure ROS production in our model. DCFH-DA (20 µM) was added for 30 minutes at 37°C in the dark after stimulation. Intracellular ROS levels was measured in a fluorometer (Kontron Instruments, Japan). For DCF fluorescence imaging, Caco-2 cells were grown for 3 days on the cover glass, then fixed and permeabilized with paraformaldeyde 4% and Triton 0,2% for 30 minutes at 4°C. DCF-HA 20 µM was added for 30 minutes at 37°C in the dark. Fluorescence images from multiple fields were obtained using a Nikon Eclipse e 80i microscopy. The images were analyzed using NiS Elements D imaging software (Nikon Instruments Inc., NY, USA).

### Glutathione Assay

GSH/GSSG ratio at intracellular level was measured by a fluorimetric assay kit (Biovision Milpitas, CA). The values were normalized for protein content and expressed as % of control.

### Transepithelial Electrical Resistance Measurements

Transepithelial electrical resistance (TEER) of cell monolayers grown on filters was measured using a Millicel-ERS resistance monitoring apparatus (Millipore). The net TEER (in Ohms/cm^2^) was calculated by subtracting the background from the actual value and multiplying the value obtained by the area of the filter (4.9 cm^2^).

### Immunofluorescence Methods

Actin staining: fixation and permeabilization was performed with 4% paraformaldehyde and 0.2% Triton X-100 for 30 min at + 4°C. Cells were treated with 50 µg/ml solution of Alexa Fluor 594-phalloidin (Sigma-Aldrich) for 40 min.

Occludin staining: cells were fixed by adding 100% methanol for 10 min at room temperature and probed with anti-occludin antibody (Abcam ab59720) over night at +4°C. Bound antibody was detected with Alexa fluor 488 conjugated anti-rabbit IgG antibody (Invitrogen, A21206).

Immunofluorescence assay for NF-kB: Caco-2 cells were grown on the chambered cover glass for 3 days, fixed with 4% buffered paraformaldehyde (pH 7.4) for 30 minutes followed by blocking and permeabilization for 1 h in PBS with 1% BSA and 0.1% Triton X-100 at room temperature. Caco-2 cells were then incubated at 4°C with anti-NFkB p65 antibody (Santa Cruz Biotechnology, Santa Cruz, CA, USA) in a humidified chamber and incubated with a FITC-conjugated goat anti-rabbit antibody at room temperature and with Hoechst 33342 (10 µg/mL) for 5 min.

For all immunofluorescence studies, slides were mounted with Vectashield Mounting Medium with DAPI (Vector laboratories, Ltd, UK). Fluorescence images from multiple fields were obtained using a Nikon Eclipse e 80i microscopy. The images were analyzed using NiS Elements D imaging software (Nikon Instruments Inc., NY, USA).

### Caspase-3 Activity Assay

We used caspase-3 as a marker of apoptosis (Furuya-Kanamori et al., 2015). An apoptosis assay kit was used to determine caspase-3 activity, according to the manufacturer’s instructions (Biovision, Mountain View, CA). Caspase-3 activity was investigated in Caco-2 cells by the release of the chromophore pNA after substrate cleavage. Modifications of caspase-3 activity were determined by comparing the sample optical density (OD) with the control.

### Immunoblotting

Total cell lysates were obtained by homogenization of cell pellets in cold lysis buffer (20 mM Tris, pH 7.5 containing 300 mM sucrose, 60 mM KCl, 15 mM NaC1, 5% (v/v) glycerol, 2 mM EDTA, 1% (v/v) Triton X-100, 1 mM PMSF, 2 mg/ml aprotinin, 2 mg/ml leupeptin and 0.2% (w/v) deoxycholate) for 1 min at 4°C and then sonication for 30 sec at 4°C. Equal amounts of protein were separated on 10% (v/v) SDS-PAGE and transferred to a PVDF membrane (Millipore). The membrane was blocked with 5% (w/v) skim milk. Then, incubation with primary antibody followed by an HRP-conjugated secondary antibody were performed. Separated proteins were visualized with the ECL detection system (GE-Healthcare). The following antibodies were used for Western blot analysis: anti-caspase-3, anti-NFkB p65 and anti-Tubulin antibodies (Santa Cruz Biotechnology).

### Statistical Analysis

GraphPad Prism Software (San Diego, CA) was used to evaluate the two-tailed unpaired Student t test. In addition, a 2-tailed paired Student t test to evaluate statistical significance. The alpha value of 0.05 was set for statistical significance. p-values for each analysis are indicated in figure legends.

## Results

### Effects of LGG on RV-Induced Enterotoxic Effect

**Figure 1:**
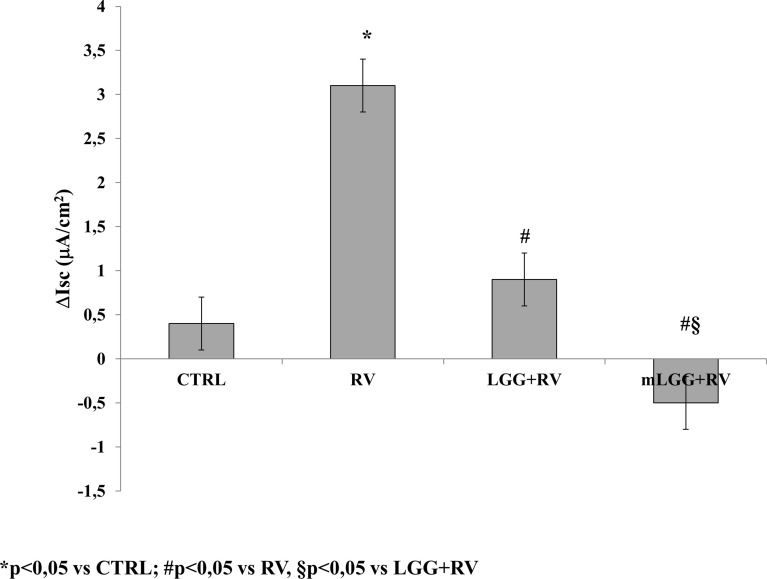
Effects of LGG on enterotoxic effect induced by RV. Caco-2 cell monolayers were infected with RV and preincubated with LGG or mLGG as described in the Methods and then, the short-circuit current (*I*sc) was evaluated in Ussing chambers togheter with unifected cells (CTRL). *p < 0.05 vs CTRL; ^#^p < 0.05 vs RV; ^§^p < 0.05 vs LGG+RV.

As previously reported in the basic model of Caco-2 cells, RV-induced chloride secretion is NSP4-dependent and involves oxidative stress (De Marco et al., 2009; Buccigrossi et al., 2014). To investigate the effects of LGG on RV-induced chloride secretion, we preincubated Caco-2 cell monolayers with living LGG (LGG) or the conditioned medium from LGG (mLGG) before RV infection. In Ussing chamber experiments, the magnitude of short circuit current (Δ*Isc*), which reflects the intensity of RV-induced chloride secretion, was significantly reduced by LGG and completely abolished by mLGG (**Figure 1**). In the latter condition, a pro-absorptive effect was also observed. LGG and mLGG alone did not induce *Isc* changes (data not shown).

**Figure 2:**
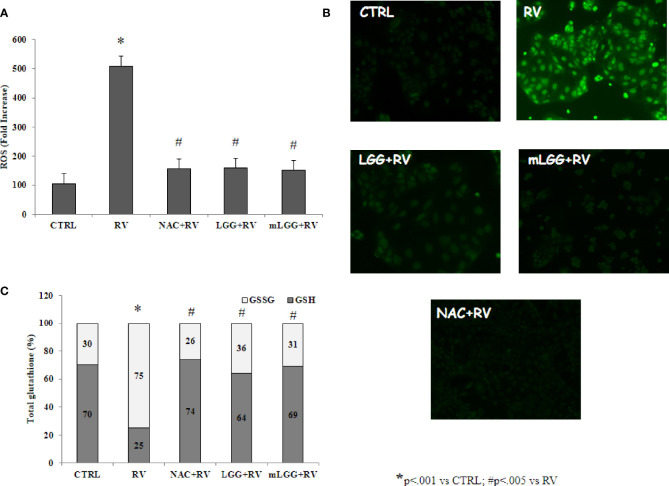
Effect of LGG on oxidative stress induced by RV. **(A)** ROS production was evaluated in Caco-2 cell monolayers infected with RV preincubated with LGG or mLGG as described in the Methods. [*p< 0.05 vs CTRL; #p<0,05 vs RV]. **(B)** Caco2 cells were infected with RV following preincubation in the absence of LGG or mLGG and the fluorescence of the ROS probe was evaluated one hour following infection. Magnification: 400X **(C)** Caco-2 cells monolayers were infected with RV and glutathione was evaluated one hour following infection and levels of GSH (gray) and GSSG (white) were measured. LGG and mLGG preincubation are present during the activation phase of the virus as described in the Methods. [*p < 0.05 vs control; ^#^p < 0.05 vs RV]. In all experiments, NAC was used as antioxidant factor.

Since RV-induced enterotoxic damage is oxidative stress-dependent (De Marco et al., 2009), the redox state was evaluated in Caco-2 cells infected with RV following preincubation with LGG or mLGG. RV induced ROS production that was completely inhibited by LGG and mLGG at the same levels of N-acetylcysteine (NAC), a potent antioxidant (**Figures 2A, B**). To evaluate the role of antioxidant defenses, we determined the levels of reduced and oxidized glutathione (GSH/GSSG ratio) in RV-infected Caco-2 cells following preincubation with LGG or mLGG. The GSH/GSSG ratio was maintained when RV-infected cells were exposed to LGG or mLGG (**Figure 2C**). In all experiments, NAC was used as antioxidant factor. Our results indicated that NAC preincubation results in an anti-oxidant preventive effect on ROS production and GSH/GSSG ratio (**Figure 2**).

Since NSP4 induces chloride secretion by Ca^2+^ pathways, we investigated the role of Ca^2+^ in our model. Thapsigargin, an inhibitor of the ER Ca^2+^-ATPase (Michelangeli et al., 1995; Díaz et al., 2012), selectively depletes the endoplasmic reticulum Ca^2+^ stores. Thapsigargin induced an increase in *Isc* (0.9 ± 0.5 vs 16.1 ± 1.5; p<0.05) but no difference was found when cells were preincubated with LGG or mLGG (control 16.1 ± 1.5; LGG 14.9 ± 0.9; mLGG 13.7 ± 1.2; p=NS) suggesting that the mechanism of action is upstream Ca^2+^ release.

### Effects of LGG on RV-Induced Cytotoxic Damage

To investigate LGG effects on RV-induced epithelial damage, RV infected Caco-2 cell monolayers were exposed to LGG or mLGG and intestinal epithelial integrity was evaluated.

**Figure 3:**
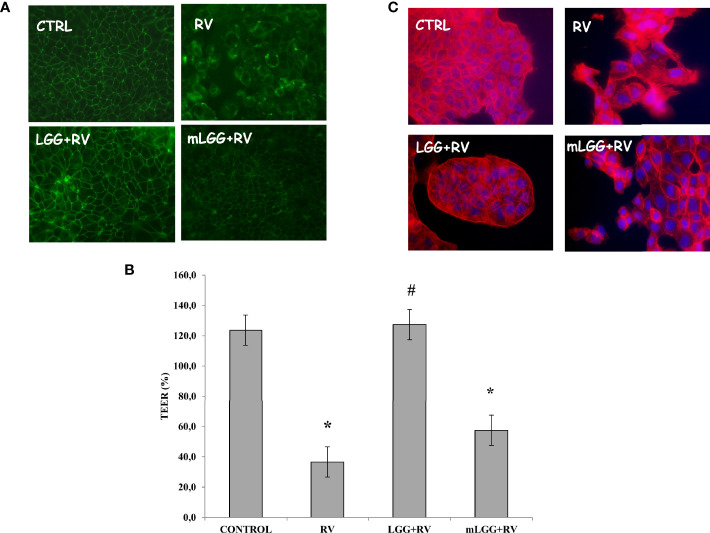
Effect of LGG on cytotoxic damage induced by RV. **(A)** Occludin, as marker of tight junction structure, was evaluated with immunofluorescence in Caco-2 cell monolayers infected with RV preincubated with LGG or mLGG as described in the Methods Magnification: 400X. **(B)** Caco-2 cells monolayers were infected with RV following preincubation in the of LGG or mLGG and transepithelial electrical resistence (TEER) was evaluated as described in the Methods together with uninfected cells; [*p < 0.05 vs CTRL; ^#^p < 0.05 vs RV]. **(C)** Phalloidin, as marker of cytoskeleton architecture, was used with immunofluorescence in Caco-2 cell monolayers infected with RV preincubated with LGG or mLGG as described in the Methods Magnification: 400X.

Occludin staining looked like a network in untreated and well differentiated cells whereas RV induces a major break of the junctions between the cells. The presence of LGG, but not mLGG, preserved cells from RV-induced damage (**Figure 3A**). This effect was also confirmed by transepithelial resistance data. RV induced a strong decrease of TEER that was significantly prevented by LGG but not by mLGG (**Figure 3B**). Finally, actin staining also showed that LGG living microorganisms but not mLGG, protects cells by cytoskeleton destruction induced by RV (**Figure 3C**).

### Effects of LGG on RV-Induced Apoptosis

**Figure 4:**
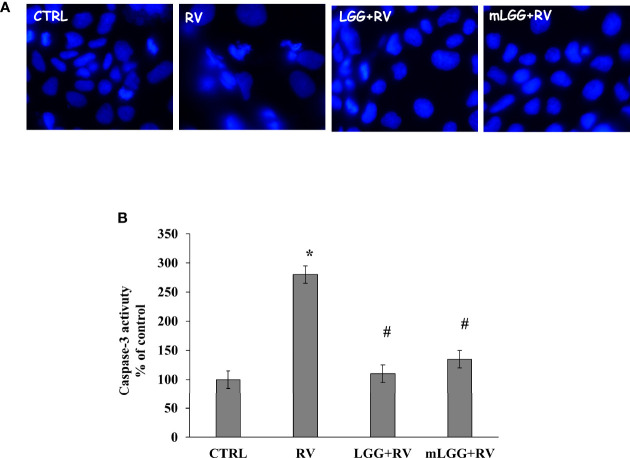
Effect of LGG on apoptosis induced by RV. **(A)** Apoptotic nuclei were evaluated in Caco-2 cell monolayers infected with RV preincubated with LGG or mLGG as described in the Methods Magnification: 400X. **(B)** Caco2 cells were infected with RV following preincubation in the absence of LGG or mLGG and caspase-3 activity as apoptotic marker was evaluated as described in Methds [*p < 0.05 vs CTRL; ^#^p < 0.05 vs RV].

Apoptosis was evaluated by nuclei staining and caspase activation. RV infected cells have smaller nuclei with irregular edges (**Figure 4A**). Because DAPI staining is an indirect marker of apoptosis, caspase-3 activation was evaluated. We observed that RV infection increases caspase-3 activity suggesting that apoptosis is one of the mechanisms of cell damage. Both LGG and mLGG significantly reduced apoptosis induced by RV with a similar efficacy (**Figures 4A, B**).

### Effects of LGG on RV-Induced NF-kB Activation

NF-kB activation has a key role in intestinal epithelial permeability (Dominguez et al., 2013; Yang et al., 2014) as well as in infections (Santoro et al., 2003), including RV (Rossen et al., 2004) and SARS-CoV-2 (Poeta et al., 2021) infections. NF-kB is found in the cytoplasm as an inactive factor, a heterodimer of p50 and p65 subunits bound with a member of the IkB inhibitor protein family. Removal of IkB, induced by several stimuli, results in NF-kB activation through p65 phosphorylation and its translocation to the nucleus (Campbell and Perkins, 2006). To see whether NF-kB was activated in response to RV infection, we evaluated the levels of phospho-p65 and its nuclear localization in RV-infected Caco-2 cells.

**Figure 5:**
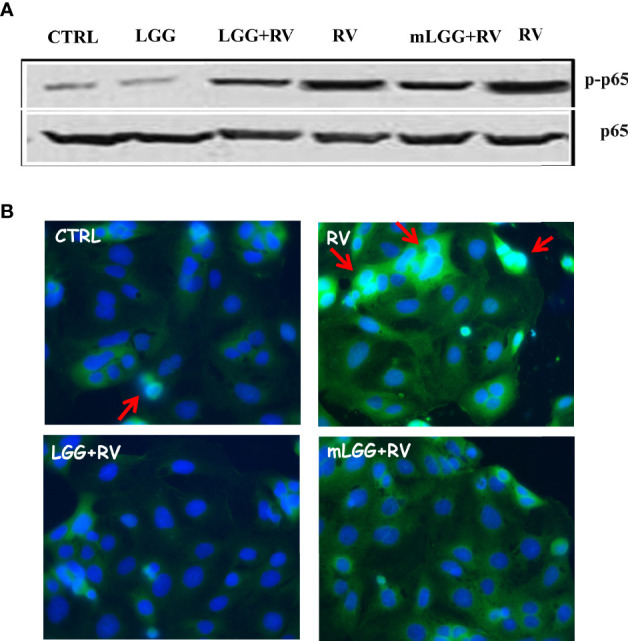
Effect of LGG on RV-induced activation of NF-kB pathway in Caco-2 cells. **(A)** Activated NF-kB p65 subunit (upper panel) was evaluated in RV-infected Caco-2 cells with or without the addition of mLGG or LGG and compared to total p65 levels in a western blot experiment. **(B)** NF-kB p65 subunit was detected in RV-infected cells with or without the addition of mLGG or LGG with immunofluorescent method as described in “Method” section and nuclei were stained by Hoerst. Red arrows indicate the p65 nuclear localization. Data are representative of 3 separate experiments.

As shown in **Figure 5A**, the levels of phospho-p65 were increased in cell extracts following RV infection. We also observed p65 translocation from cytoplasm to the nucleus with immunofluorescence (**Figure 5B**). Phospho-p65 intracellular levels was inhibited in RV-infected cells exposed to LGG and mLGG with a similar efficacy (**Figure 5A**) and the nuclear translocation did not ensue (**Figure 5B**).

## Discussion

Our data demonstrated that conditioned medium of LGG has a stronger effect than living microorganisms in reducing ion secretion induced by RV. In Ussing chamber system, living LGG significantly reduced *Isc* but its conditioned medium was much stronger and even reverted the ion flux toward a proabsorbitive condition. Both preparations significantly reduced oxidative stress which is the key mechanism of ionic secretion induced by RV at intestinal level (Buccigrossi et al., 2014). Our observations are in agreement with the results of a transcriptome analysis of a recent study, which showed the downregulation of genes related to oxidative stress in Caco-2 cells treated with living LGG (Hou et al., 2019).

The cytotoxic effect by RV is observed in the subsequent phase of infection (De Marco et al., 2009). We evaluated the effects of LGG and mLGG by different approaches. Immunofluorescence technique allowed us to evaluate the tight junctions and cytoskeleton integrity by occludin and actin staining. We observed that both LGG and mLGG protect enterocytes from RV damage but the former preparation was more effective. Because the transepithelial resistance provides a sensitive quantitative measure of the effect, we could obtain compelling proof that living LGG more effectively counteracts cell damage than mLGG. However, both forms completely inhibited apoptosis and p65 activation and its translocation from cytoplasm to the nucleus thereby hampering RV pathological effects. This is in agreement with other studies in which LGG counteracted intestinal inflammation (Fong et al., 2016).

Probiotics exert antidiarrheal effects through several mechanisms of actions, including the microbiota restoration, the secretion of antimicrobial substances, the competitive exclusion of pathogens (Plaza-Diaz et al., 2020) and the modulation of the local and systemic immune response (Plaza-Díaz et al., 2018). Although these mechanisms may contribute to the antidiarrheal effect of probiotics administration, they physiologically require hours or even days to establish (Amamoto et al., 2021). However, from a clinical point of view, we previously observed that LGG administration reduces the duration of diarrhea in children with RV infection (Guarino et al., 1997) and the effect on stool outputs is observed as early as after a few hours from its administration. This effect observed over hours is not consistent with a modification of intestinal microbiota and is more likely consistent with a “pharmacological” action.

In the last 10 years, the term “postbiotic” caught interest of researchers and companies (Collado et al., 2019; Dunand et al., 2019; Rad et al., 2020), and, recently, the International Scientific Association of Probiotics and Prebiotics (ISAPP) defined postbiotics as “preparations of inanimate microorganisms and/or their components that confers a health benefit on the host” (Salminen et al., 2021). Compared with probiotics, products obtained from non-viable microorganisms are a promising alternative to improve health and counteract pathogens and drugs, being potentially “pure” and dosable. Although probiotics have an undisputed role in the prevention and treatment of several pathological conditions, there is a risk of living bacterial translocation from gut lumen to bloodstream particularly in leaky gut conditions. This implicates risk of severe clinical risk of using probiotics in fragile patients such as those with Necrotizing Enterocolitis (NEC) (Neu, 2011; Pietz, 2011). Postbiotics might provide a safer alternative being microbial components that act as pharmacologic-like molecules in terms of absorption, metabolism and excretion. In addition, higher stability and easier to standardize treatment beg key advantages for clinical use (Rad et al., 2020).

In our experimental model, the differential efficacy between living LGG and mLGG could explain the different mechanisms of actions. Specifically, secretory diarrhea could benefit from an effect mediated by molecules secreted by the probiotic with a “pharmacological” mechanism, similarly to what happens with “postbiotics” (Salminen et al., 2021). This would explain the rapidity of effectiveness observed in clinical trials. In a subsequent phase, the living probiotic protects against epithelial damage induced by RV infection with a typical probiotic mechanism, interacting with the enterocyte in a more dynamic modality.

The main limitation of our experimental model is that it represents a simplification of what occurs in human gut environment, which is common to all *in vitro* models. Further analysis will be needed to confirm our findings in *ex vivo* and *in vivo* models. In conclusion, our study contributes to a better understanding of the mechanism of efficacy of LGG in RV-induced diarrhea and contributes to identify the target mechanisms which may exploit a battery of molecules.

## Data Availability Statement

The raw data supporting the conclusions of this article will be made available by the authors, without undue reservation.

## Author Contributions

VB and AG conceived and designed the experiments. VC and ST performed the experiments. VB and AG supervised the research project. VB, MP, VC, FN, and AL analyzed the data. VB and AG wrote the paper. All authors read and approved the final manuscript.

## Funding

The study was in part supported by *Dicofarm* S.p.A. The funding sponsors had no role in the design of the study; in the collection, analyses, or interpretation of data; in the writing of the manuscript, and in the decision to publish the results.

## Conflict of Interest

The study was in part supported by Dicofarm S.p.A. The funding sponsors had no role in the design of the study; in the collection, analyses, or interpretation of data; in the writing of the manuscript, and in the decision to publish the results. All authors declare no other competing interests.

## Publisher’s Note

All claims expressed in this article are solely those of the authors and do not necessarily represent those of their affiliated organizations, or those of the publisher, the editors and the reviewers. Any product that may be evaluated in this article, or claim that may be made by its manufacturer, is not guaranteed or endorsed by the publisher.
